# Notes on the Imperial Yeomanry Base Hospital at Deelfontein

**Published:** 1900-09-22

**Authors:** L. V. Cargill

**Affiliations:** Ophthalmic Surgeon to the Hospital.


### NOTES ON THE IMPERIAL YEOMANRY BASE HOSPITAL AT DEELFONTEIN.

By L. V. Cargill, F.R.C.S., Ophthalmic Surgeon to the Hospital.

Several changes have taken place in the staff of this hospital. Messrs. Fripp and Raymond-Johnson and Dr. Saunders have left for England. Mr. Turner and Dr. Handson, with Mr. Thornton as dresser, have gone to the Mackenzie Farm branch at Cape Town, where Mr. Turner is acting as P.M.O. The medical side consists of Dr. Washbourn, senior consulting physician, and Drs. Elliot, Richmond, Breeks, and Black. Mr. Hamilton A. Ballance has become the senior consulting surgeon, the other surgeons being Messrs. Christopherson, Ashdowne, Parker, and Bruce. Mr. Cargill still remains in charge of the ophthalmic department, and Mr. Hall Edwards in charge of the *x*-ray department. The dressers are Messrs. Atkins, Blathwayt, Pearson, Ransford, Richards, Hawkins, and Sells. Dr. Elliot and Mr. Parker, who have been away on sick leave, have returned to duty. Mr. Greenfield, one of the dressers who has had enteric, has been away on sick leave to Lady Gifford's convalescent home at Norval's Pont, and is returning to England. Of the nursing staff Sisters Brereton and Leggatt have gone to the Pretoria branch, and Sisters Stephenson, Cable, and Cooper to the Mackenzie's Farm branch, Cape Town.

Under the supervision of Mr. Hall-Edwards, electric light has now been installed in the cruciform building containing the operating theatre and surgical store, *x*-ray and photographic rooms, and surgery. It is proving a great boon. The dynamo is worked by a four-horse power oil engine.

The Russian bath which has been opened in the laundry building is a great success, and is largely patronised. It is managed by Hale-Smith, the chief masseur.

There are 770 patients in hospital, 38 of whom are officers, and nearly one-half of the total number belong to the Imperial Yeomanry. Patients are being received and discharged daily, and a week ago No. 4 Ambulance Train brought 17 officers and 95 men from Winburg, most of whom came from General Clements' force. They were nearly all surgical cases. Judging from the few cases we now have of enteric and dysentery, the epidemic has completely subsided. The majority of the medical cases now admitted consist of various forms of rheumatism. As a rule, the rheumatism takes a subacute form, which is not readily amenable to treatment. Presumably its prevalence is due to the weather which, contrary to the general rule, has been not only cold but exceptionally damp, in spite of this being the dry season for up-country. In addition to rheumatism a not inconsiderable number of cases of malaria have been admitted, the patients either being old sufferers from malaria, or having contracted the disease north of Bloemfontein and Kimberley.

One of the wardmaids has developed measles, but from what source has not at present been determined. There have been no further cases of scarlet fever. Among the enteric cases there have been several cases of typhilitis, usually recrudescences of a former attack. A case recently operated on by Mr. Ballance is now doing well.

Dr. Washbourn reports that musk has been of great value in the treatment of certain cases of enteric. In a case in which the patient was comatose, and almost pulseless, rapid improvement ensued upon the administration of musk in five-grain doses every four hours. The improvement was maintained, and the patient is now convalescent. In another case of enteric, with persistent pyrexia continuing after the other symptoms had subsided, the administration of salol caused the temperature to return to normal in a couple of days. It would appear that the pyrexia was due to putrefactive changes occurring in the intestinal canal which were prevented by the salol.

In addition to the operations performed on wounded men, there has been a fair amount of ordinary civil surgery lately. During the last week Mr. Ballance has operated on two cases of mastoid disease following recent otitis media. In one case the patient was suffering from enteric, and in the other from scurvy. There are in hospital a considerable number of cases of compound fracture of the lower limb, with extensive and troublesome necrosis, needing repeated operations.

The weather here has been very variable. A few days have been genial, but other days bitterly cold, windy, and gloomy, and we have had snow and hail as well as rain. Fortunately, last Wednesday was a fine day for the athletic sports, which had been chiefly organised by Captain Mosley Leigh, of the Cheshire Yeomanry. They proved a great relief to the monotony of camp life, and were immensely appreciated by all.

We are expecting the Hospital Commission here next Thursday, and our chaplain, the Rev. J. Blackbourne, has gone to Cape Town to give evidence before them regarding his experiences as Chaplain to the Sixth Division on the march from Modder River to Bloemfontein.

